# Zinc Oxide Nanoparticles Induced Testicular Toxicity Through Inflammation and Reducing Testosterone and Cell Viability in Adult Male Rats

**DOI:** 10.1007/s12011-024-04330-1

**Published:** 2024-08-12

**Authors:** Dina H. Ahmed, Nadia M. El-Beih, Enas A. El-Hussieny, Wael M. El-Sayed

**Affiliations:** Department of Zoology, Faculty of Science, Ain Shams University, Abbassia, 11566 Cairo Egypt

**Keywords:** Apoptosis, CYP17A1, LH, Myeloperoxidase, p53, Testosterone

## Abstract

Zinc oxide nanoparticles (ZnO NPs) have wide applications in daily life. Therefore, there is growing interest in the potential harmful impacts of these particles on human health. The present study was conducted to investigate the potential toxic effects of ZnO NPs (40 and 70 nm) compared to ZnO on the testes of rats. ZnO NPs were synthesized and characterized by transmission electron microscopy (TEM) and X-ray diffraction (XRD). Adult male rats were randomly divided into four groups (*n* = 8): Group I (control), Group II (ZnO) received daily oral administration of ZnO (50 mg/kg), and Groups III and IV received daily oral administration of ZnO NPs of 40 nm or 70 nm at 50 mg/kg, respectively. All treatments continued for 50 consecutive days. ZnO and ZnO NPs reduced body and testis weights, sperm count and motility, serum luteinizing hormone (LH) and testosterone levels, testicular cytochrome p450 17A1 (CYP17A1) and cytochrome p450 1B1 (CYP1B1) concentrations, and the expression of *p53* and *cdk1*. These treatments elevated testicular myeloperoxidase and serum acid phosphatase activities as well as sperm abnormalities. ZnO NPs reduced LH levels, which decreased CYP17A1 and CYP1B1, resulting in reduced synthesis of testosterone. ZnO NPs enhanced testicular inflammation and reduced cell viability. All these effects were manifested as reduced sperm motility and increased sperm deformities. Compared to macromolecules, nanoparticles exhibited significantly higher toxicity. The larger diameter ZnO NPs had more profound toxicity than the smaller-sized particles.

**Supplementary Information:**

The online version contains supplementary material available at 10.1007/s12011-024-04330-1.

## Introduction

Nanotechnology has rapidly advanced in various fields such as medicine, manufacturing, agriculture, food, and cosmetics in recent years. Nanoparticles (NPs), with their nanoscale size (1–100 nm) and high surface-to-volume ratio compared to bulk materials, possess unique characteristics [1]. They can enter the body through inhalation, ingestion, or skin contact, overcoming biological barriers to affect organs, including the reproductive system. Studies in mice show that NPs, including zinc oxide nanoparticles (ZnO NPs), can accumulate in tissues like the brain and testes, crossing barriers like the blood–brain and blood-testis barriers [2]. Currently, over 2,000 nanoparticle-containing products are available on the market, spanning industries such as electronics, food, sports, textiles, and antibiotics, with this number continually increasing [3].

Metal nanoparticles like ZnO NPs have gained significant attention in biomedicine and industry due to their distinctive thermal, magnetic, optical, and biological properties. They are widely used in food additives, medications, and cosmetics for their antibacterial and UV absorption capabilities [4]. However, widespread applications of ZnO NPs increase the risk of ingestion exposure [5], potentially leading to their accumulation in organs and posing health hazards [6]. Therefore, it is crucial to assess the potential health consequences of exposure to ZnO NPs, which have demonstrated cytotoxic and pro-inflammatory effects across various cell types [7]. These effects include membrane damage, inflammation, DNA damage, apoptosis, and hormonal disturbances in mammalian cells [8], attributed to the production of reactive oxygen species (ROS) [9].

Assessing the risks of exposure to ZnO NPs is particularly pertinent in fields where occupational exposure is common, such as male fertility. However, existing literature lacks consensus on the detrimental effects of these nanoparticles, often due to variations in NP size or duration of exposure. Therefore, this study investigates the toxic effects of ZnO NPs of two different sizes (40 and 70 nm) compared to bulk ZnO on the physiology and biochemical activity of the male reproductive system in rats. Our study examines parameters including sex hormones, semen characteristics, oxidative stress markers, enzymes like CYP17A1 and CYP1B1, myeloperoxidase (MPO) activity, caspase 3 levels, and the expression of *p53* and cyclin-dependent kinase 1 (*cdk1*).

## Materials and Methods

### Chemicals

Zinc oxide (ZnO) was purchased from El Naser for Intermediate Chemicals (Cairo, Egypt) and stored at a temperature below 30 °C in a dark and dry place. ZnO NPs (~ 40 and 70 nm) were synthesized by sol–gel method as described elsewhere [10]. The powder was white in color, and the particles were spherical.

### Characterization of ZnO NPs

**Fig. 1:**
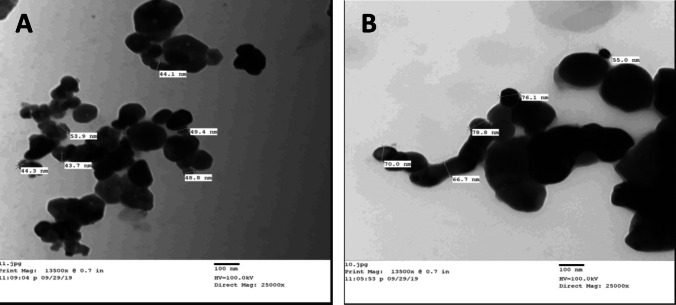
Transmission electron microscope (TEM) of ZnO nanoparticles (**A**) small diameter (⁓40 nm), and (**B**) large diameter (⁓70 nm)

**Fig. 2:**
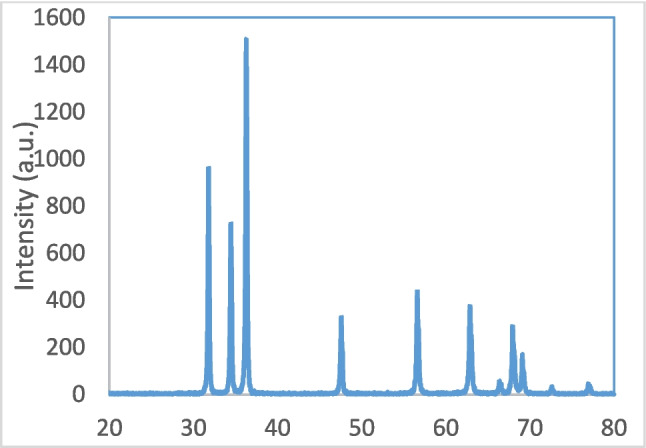
X-ray diffraction (XRD) pattern of ZnO nanoparticles

Transmission electron microscopy (TEM) was used to determine the morphological characteristics of the ZnO NPs at the National Research Center (Cairo, Egypt). The NPs were dispersed in distilled water, ultrasonicated for about 15 min, deposited on copper grids, dried, and then examined by TEM. The size of the nanoparticles was confirmed by TEM (Fig. 1) and X-ray diffraction (XRD) (Fig. 2).

### Animals

A total of thirty-two adult healthy male rats (*Rattus norvegicus*), weighing 140–150 g at the beginning of the experiment, were obtained from the Veterinary Serum and Vaccine Research Institute (Abbassia, Cairo, Egypt). They were transported to the animal house at the Department of Zoology, Faculty of Science, Ain Shams University. The animals were housed in appropriate cages (4 rats per cage) and provided with ad libitum access to water and standard rodent food pellets. The food pellets were purchased from El Hayani Co. (Al-Tal Al-Kabir, Ismailia – Egypt). The diet ingredients are shown in Table S1 (See the supplementary). Prior to the experiment, the animals were acclimatized for one week. Weekly weighing and observation for any symptoms were conducted and recorded. All animals received humane care, and all procedures and experimental protocols were conducted following the ARRIVE guidelines. The research protocol was approved by the Ethics Committee of the Faculty of Science, Ain Shams University, Egypt (ASU-SCI/ZOOL/2024/4/10).

### Experimental Design

The experimental animals were randomly divided into four groups (*n* = 8). Group I (Control): the animals received a daily oral administration of distilled water. Group II (ZnO): the animals received a daily oral administration of ZnO (50 mg/kg) [11]. Groups III and IV: the animals received a daily oral administration of ZnO NPs 40 nm or 70 nm at 50 mg/kg, respectively [12]. The duration from initiation of stem cell division to formation of mature spermatozoa is 50 days [13]. Therefore, the treatments continued for 50 consecutive days.

### Body and Testis Weights

The body weights of the rats were recorded weekly throughout the experimental period (7 weeks), followed by the calculation of body weight changes and the relative testis weight after dissection.

### Blood and Tissue Sampling

At the end of the experiment, the animals were fasted overnight, anesthetized with isoflurane, and sacrificed. Blood was collected into clean test tubes and left to coagulate at room temperature, then centrifuged at 4000 rpm for 10 min at 4 °C. Serum was immediately separated, divided into aliquots, and stored at -80 °C until used for the determination of free testosterone, follicle-stimulating hormone (FSH), luteinizing hormone (LH), alanine aminotransferase (ALT), and acid phosphatase. The testes were excised, cleared of adhering connective tissue, rinsed thoroughly in sterile isotonic saline, blotted dry with filter paper, and weighed. Testicular tissue was then divided into different aliquots and preserved at -80 °C for further analyses. For molecular investigations, testicular aliquots were preserved in TRIzol (1 ml for each 100 mg of tissue).

### Sperm Analyses

The cauda epididymis and upper portions of the vas deferens of rats were cleaned to remove adherent tissue. Subsequently, they were placed in a plate containing isotonic saline to wash out any blood. The cauda epididymis was then longitudinally cut into pieces to release sperms into a Petri dish containing 2 ml of isotonic saline at 37 °C. The sperm suspensions were incubated for 10 min at 37 °C to ensure complete release of spermatozoa from the cauda epididymis into the saline solution.

The sperm count, motility, and morphology of sperms were performed as described elsewhere [14, 15]. Briefly, sperms were counted using the hemocytometer in the five squares under the light microscope at 40X, ignoring the cells on the upper and right square boundaries. Sperms were diluted 10 times with a buffer. A volume of 10 µl of the diluted sperm suspension was placed under a cover slip of a hemocytometer. Sperm motility was determined by scoring the number of motile sperms (the strongest and fastest swimming sperms on straight lines), non-progressive motile (sperms move forward but tend to travel in a curved or crooked motion), and immotile sperms (these are failing to move at all) in the same field using a hemocytometer under a light microscope. For morphological examination of the sperms, a sperm smear on a slide was prepared, air-dried, and fixed in absolute methanol. The slides were then stained with 1% aqueous Eosin-Y solution for an hour. The slides were washed with distilled water, passed through neutral resin, and mounted with coverslips.

### Biochemical Assays in Serum

The levels of free testosterone, FSH, and LH in serum were measured using sandwich ELISA. Kits for free testosterone and FSH were purchased from Bioactive Diagnostic (GmbH, Louisenstr, Bad Homburg), with catalog numbers BDFTT11-BA and BDFS08-BA, respectively. The LH kit was obtained from PerkinElmer Health Sciences (Massachusetts, USA), catalog number 10004. Alanine aminotransferase (ALT) activity was assessed using a colorimetric kit provided by Spectrum (Cairo, Egypt), catalog number 264002. Acid phosphatase activity was determined using a colorimetric kit from Bio Diagnostic (Giza, Egypt), Cat # AC 1010.

### Biochemical Assays in the Testis

#### Oxidative Stress Markers

Using an electric homogenizer (Universal Laboratory Aid MPW-309, Poland), the testis tissue was homogenized in ice-cold phosphate-buffered saline (PBS), pH 7.4. The homogenates were centrifuged at 18,000 × g for 20 min at 4 °C using a Cooling Microfuge Laborzentrifugen (Sigma, Germany). The supernatants were then collected, aliquoted, and stored at -80 °C until analysis. This homogenate was used for the determination of oxidative stress parameters including malondialdehyde (MDA), reduced glutathione (GSH), superoxide dismutase (SOD), and total protein content. Tissue protein content was determined as described before [16]. SOD activity was estimated as described elsewhere [17]. GSH and MDA were determined as described elsewhere [18, 19], respectively.

#### Assessment of Cytochrome P540 (CYP17A1 and CYP1B1)

The CYP17A1 concentration was estimated in the testis homogenate by Western blot. The primary antibodies against CYP17A1 and β-actin were purchased from Proteintech Group (USA), catalog numbers 14447–1-AP and Abcam plc (USA), catalog number ab8227, respectively. The anti-Rabbit IgG-horseradish peroxidase antibody produced in goat was obtained from Sigma (St. Louis, MO, USA), catalog number A6154. The procedures for lysis, membrane transfer, washing, detection, and visualization can be found in the supplementary file and were performed according to established protocols [20]. GraphPad Prism 8 software was used to calculate the fold change in protein level after normalizing to the concentration of the housekeeping protein β-actin. The CYP1B1 concentration was estimated in the testis homogenate using a sandwich ELISA kit provided by Creative Diagnostics (Ramsey Road, Shirley, USA), catalog number DEIA-FN364.

#### Assessment of Myeloperoxidase (MPO) Concentration and Caspase-3 Activity

MPO concentration was assayed in the testis homogenate using a sandwich ELISA kit provided by Biovision (Milpitas, USA), catalog number E4581-100. The activity of caspase-3 was determined using the caspase-3/CPP32 colorimetric assay kit [21].

#### Assessment of Zinc Bioaccumulation in Testis

The concentration of zinc in the testicular tissue was assayed using a Microwave Digestion System (MDS-2000) from CEM, USA. This method involves nitric acid digestion of rat testes in a closed vessel under pressure-controlled microwave heating for the determination of zinc using spectroscopic methods.

#### Determination of the Relative Expression of *cdk1 *and *p53* by Quantitative Real-time Polymerase Chain Reaction (q-RT-PCR)

Total RNA was extracted from the testes using TRIzol reagent purchased from Bioer Technology Co. Ltd. (Binjiang District, Hangzhou, China). cDNA synthesis and qPCR were performed using specific primers for *p53, cdk1,* and glyceraldehyde-3-phosphate dehydrogenase *(GAPDH)* as a housekeeping gene. All primers (Table 1) were obtained from Sigma (St. Louis, MO, USA).

**Table 1:** Sequence of primers of the investigated genes and the housekeeping gene

Gene	Primer sequence (5’-3’)	GC%	Tm ºC	Accession no
*p53*	F: GATTCCTGCTTCCTTCAGTT R: CCACAGCTAGCTTCACTGAG	45.00 55.00	60.8 63.1	24842
*Cdk1*	F: ACAGAGAGGGTCCGTTGT R: CGTACTGGGCACTCCTTCTT	50.00 50.00	61ºC 62.1	54237
*GAPDH*	F: CTCCCATTCTTCCACCTTTG R: CTTGCTCTCAGTATCCTTGC	50.00 50.00	61.4 61.1	24383

### Statistical Analysis

The statistical analysis was performed using the Statistical Package for the Social Sciences (SPSS) version 16 for Windows. The distribution of the data was assessed using the Kolmogorov–Smirnov test. Tukey's test was used for multiple comparisons following One-Way Analysis of Variance (ANOVA). The data were expressed as mean ± standard error of the mean (SEM), and statistical significance was determined at *P* < 0.05.

## Results

### Body Weight Measurements

**Fig. 3:**
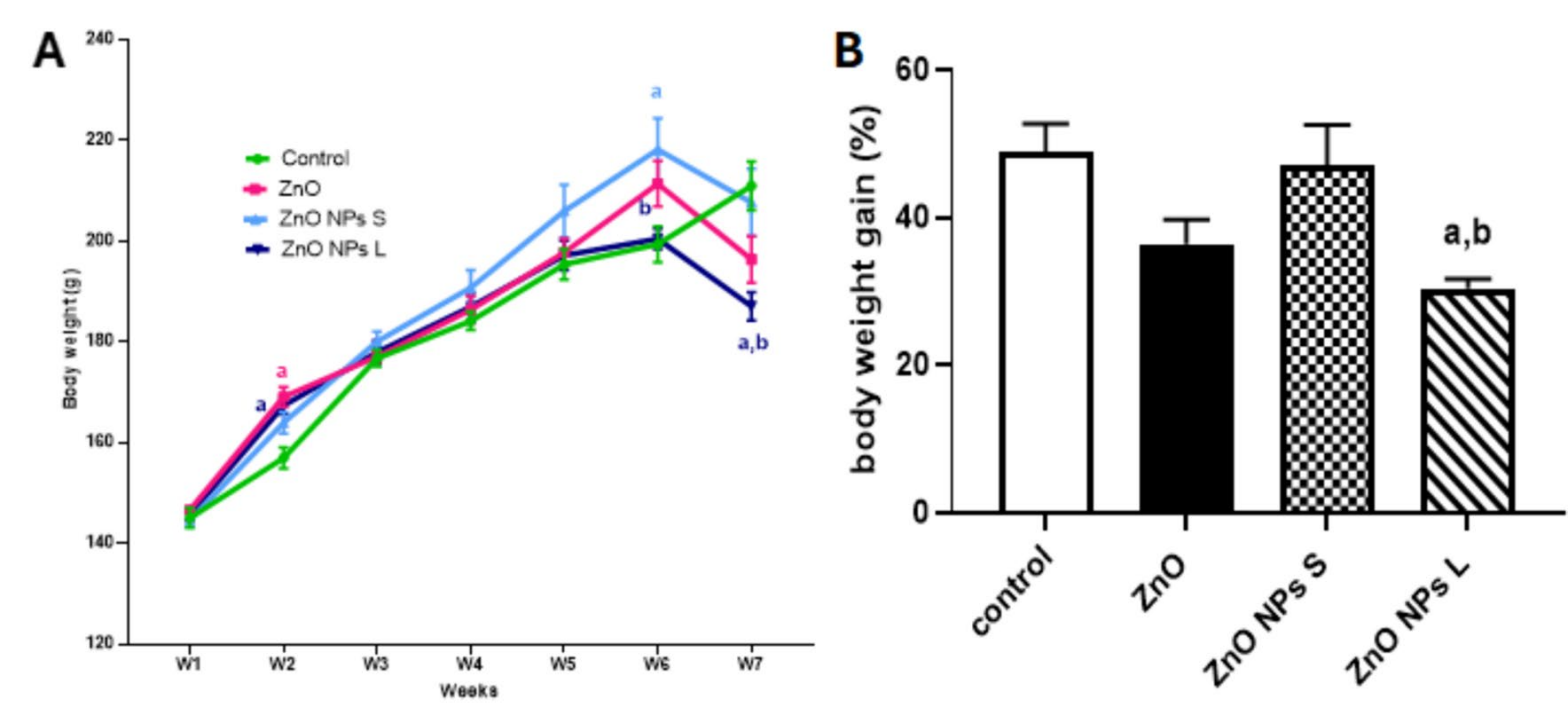
(**A**) Mean body weight throughout the experimental period (7 weeks), and (**B**) Body weight gain (%). Data are represented as Mean ± SEM, *n* = 8. ^a^ Significant difference versus control, ^b^ significant difference between different diameters. S: small size, L: large size

The body weight change was monitored weekly throughout the treatment period. Variations were observed between groups receiving different sizes of ZnO NPs. In the 6th week, the group treated with small-sized ZnO NPs (40 nm) exhibited a significant increase in body weight compared to the control group. The largest reduction in body weight was observed in the 7th week in the group treated with large-sized ZnO NPs (Fig. 3A). Additionally, the group treated with large-sized ZnO NPs showed a significant decrease in the percentage of body weight gain compared to both the control and the group treated with small-sized ZnO NPs (Fig. 3B).

### The relative testes weight and zinc concentration in the testes

Treating rats with ZnO significantly (*p* < 0.05) decreased the relative weight of testes by 14.3% compared to the control. Treatments with ZnO NPs of small or large diameter did not result in significant changes in the relative testes weight (Table 2). The zinc concentration in the testes was not affected by any treatment (Table 2).

**Table 2:** Effect of zinc oxide on the relative weight of testes and zinc bioaccumulation in the testes

	Control	ZnO	ZnO NPs small	ZnO NPs large
Relative testes weight	0.014 ± 0.0003	0.012 ± 0.0007^a^	0.014 ± 0.0004	0.015 ± 0.0003
Zn concentration	34.71 ± 3.62	34.29 ± 3.25	33.75 ± 3.17	37.50 ± 3.42

Results are expressed as mean ± SEM, *n* = 8. ^**a**^: significant difference versus control

### Sperm Analysis

**Fig. 4:**
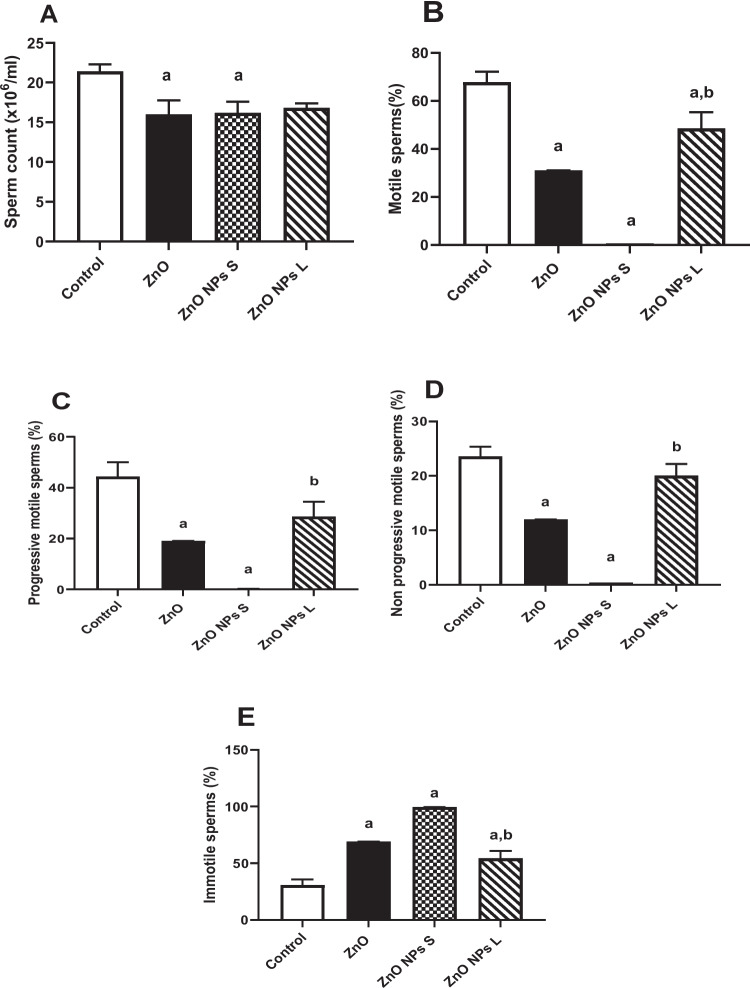
Effect of zinc oxide on sperm analysis. (**A**) Sperm count, (**B**) Motile sperm%, (**C**) progressive motile sperms%, (**D**) Non-progressive motile sperms%, and (**E**) Immotile sperms%. Data are represented as Mean ± SEM, *n* = 8, ^a^: significant difference versus control ^b^: significant difference between different diameters. S: small size and L: large size

**Fig. 5:**
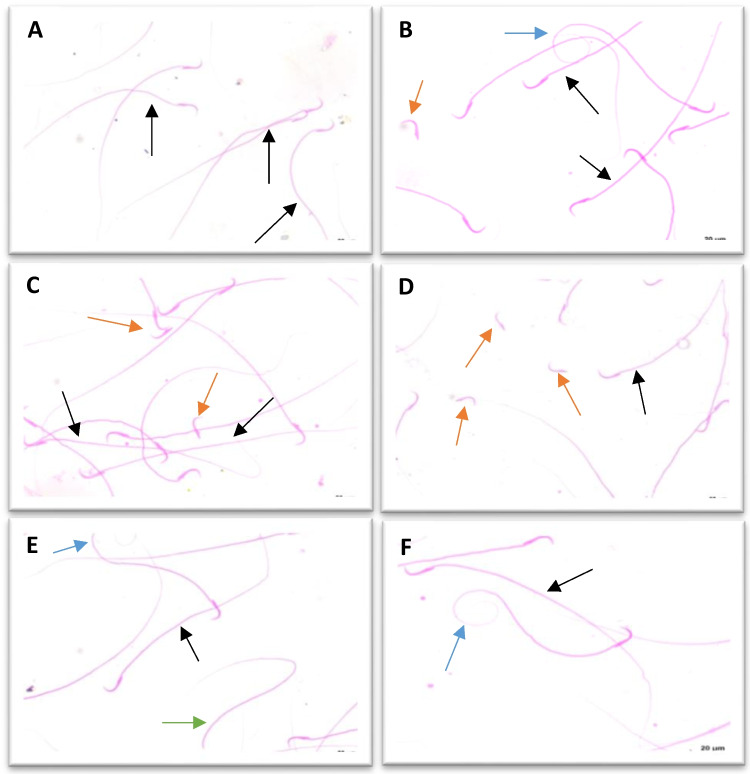
Photomicrographs of epididymal sperm smears stained with Eosin-Y. (**A**) control, (**B**) ZnO, (**C**) ZnO NPs large size, (**D**), (**E**), and (**F**) ZnO NPs small size. Morphological classification of rat epididymal sperm. Normal sperm (black arrow), tailless head (red arrow), headless tail (green arrow), and U- shaped tail (blue arrow)

Treatment of animals with ZnO and ZnO NPs of small size caused significant (*p* < 0.05) reductions in sperm count by 25% and 24%, respectively, compared to the control. ZnO NPs of larger size resulted in a 20% reduction in sperm count, but this difference did not reach statistical significance (*p* = 0.053). Treating animals with ZnO led to significant (*p* < 0.001) decreases in motile sperm (54.3%), including progressive (*p* < 0.01; 57.1%) and non-progressive (*p* < 0.001; 49.1%) sperms, compared to the control. Immotile sperms showed a significant (*p* < 0.001) increase (114.7%) compared to the control. Treating animals with ZnO NPs of small size resulted in significant (*p* < 0.001) decreases in motile sperm (99.1%), including progressive (*p* < 0.001; 99.4%) and non-progressive (*p* < 0.001; 98.4%) sperms, compared to the control. Immotile sperms showed a significant (*p* < 0.001) increase (209.2%) compared to the control (Fig. 4). Treating animals with ZnO NPs of large size led to a significant (*p* < 0.05) decrease (28.4%) in motile sperm compared to the control. Immotile sperms showed a significant (*p* < 0.01) increase (60.0%) compared to the control. Moreover, ZnO NPs of large size showed a significant (*p* < 0.001) elevation (7609%) in immotile sperm, including progressive (*p* < 0.001; 11,328%) and non-progressive (*p* < 0.001; 5163%) sperms, compared to ZnO NPs of small size (Fig. 4). Sperm morphological abnormalities induced by ZnO and ZnO NPs included tailless, headless, and U-shaped tail sperms (Fig. 5).

### The Male Sex Hormones

**Fig. 6:**
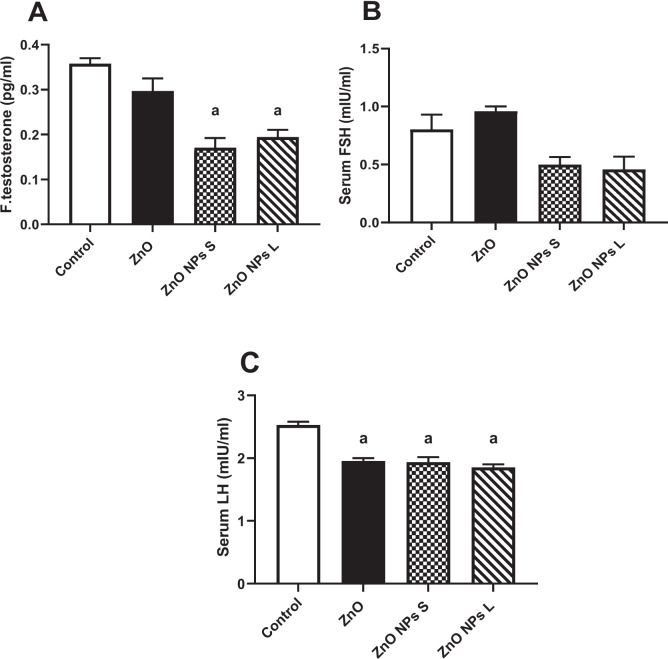
Effect of zinc oxide on the levels of male sex hormones in serum, (**A**) Free testosterone, (**B**) follicle stimulating hormone (FSH), and (**C**) luteinizing hormone (LH). Data are expressed as Mean ± SEM., *n* = 8, ^a^ significant difference versus control, ^b^ significant difference between different diameters. S: small size, L: large size

Treating rats with ZnO NPs of small and large sizes significantly (*p* < 0.001) decreased the free testosterone level in serum by 52.3% and 45.7%, respectively, compared to the control (Fig. 6A). None of the treatments caused any significant change in the serum level of FSH (Fig. 6B). The groups treated with ZnO, ZnO NPs of small size, and ZnO NPs of large size showed significant (*p* < 0.001) reductions in serum LH level by 22.7%, 23.3%, and 26.8%, respectively, compared to the control (Fig. 6C).

### Alanine Aminotransferase and Acid Phosphatase Activities in Serum

None of the treatments caused any significant change in the serum activity of ALT (Table 3). Treating animals with ZnO resulted in a significant increase in the activity of acid phosphatase in serum (*p* < 0.01; 135.7%) compared to the control. Treating animals with ZnO NPs of small size significantly elevated the enzyme activity (*p* < 0.001; 197.0%) compared to the control. Treating rats with the large size nanoparticles significantly elevated the enzyme activity by 417.8% compared to the control and by 74.4% compared to the small size group (*p* < 0.001) (Table 3).

**Table 3:** Effect of zinc oxide on serum alanine aminotransferase (ALT) and acid phosphatase activities

	Control	ZnO	ZnO NPs small size	ZnO NPs large size
ALT (U/L)	6.69 ± 0.40	9.11 ± 0.75	5.41 ± 0.47	5.60 ± 1.17
Acid phosphatase (U/L)	3.36 ± 0.24	7.92 ± 0.87^a^	9.98 ± 0.55^a^	17.4 ± 0.67^a,b^

Results are expressed as mean ± SEM, *n* = 8, ^a^: significant difference versus control ^b^: significant difference between different diameters

### Oxidative Stress Markers Assay

**Fig. 7:**
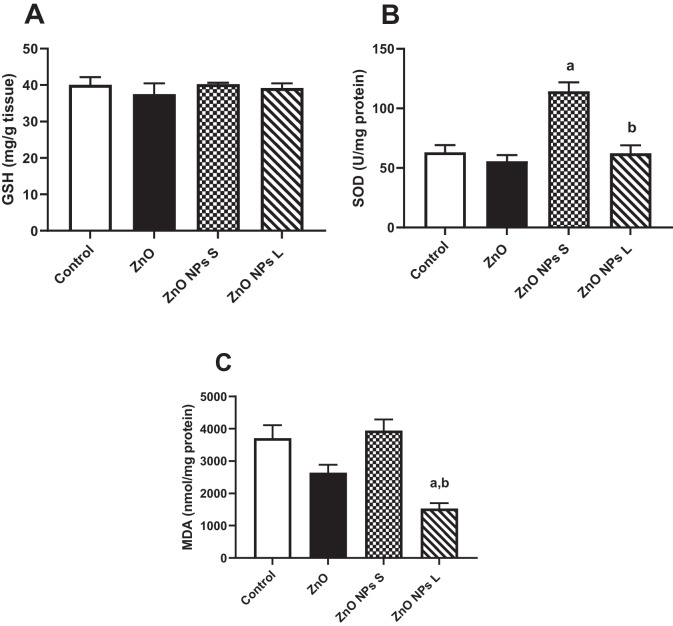
Effect of zinc oxide on oxidative stress markers in testicular tissue (**A**) reduced glutathione (GSH), (**B**) superoxide dismutase (SOD), and (**C**) malondialdehyde (MDA). Data are expressed as Mean ± SEM, *n* = 8. ^a^ Significant difference versus control, ^b^ significant difference between different diameters. S: small size and L: large size

All treatments with zinc oxide, whether at the macro or nano size, resulted in no significant changes in the reduced glutathione (GSH) level in testis homogenate (Fig. 7A). Treating animals with ZnO NPs of small size showed a significant (*p* < 0.001) elevation (80.8%) in the activity of superoxide dismutase (SOD) in the testis homogenate compared to the control (Fig. 7B). Treating animals with ZnO NPs of large size significantly (*p* < 0.001) decreased the level of malondialdehyde (MDA) in the testis by 58.7% compared to the control and by 61.2% compared to the ZnO NPs small size group (Fig. 7C).

### Cytochrome P450 17A1 (CYP17A1) Level, Cytochrome P450 1B1 (CYP1B1) Activity, and Myeloperoxidase (MPO) Activity in the Testis

**Fig. 8:**
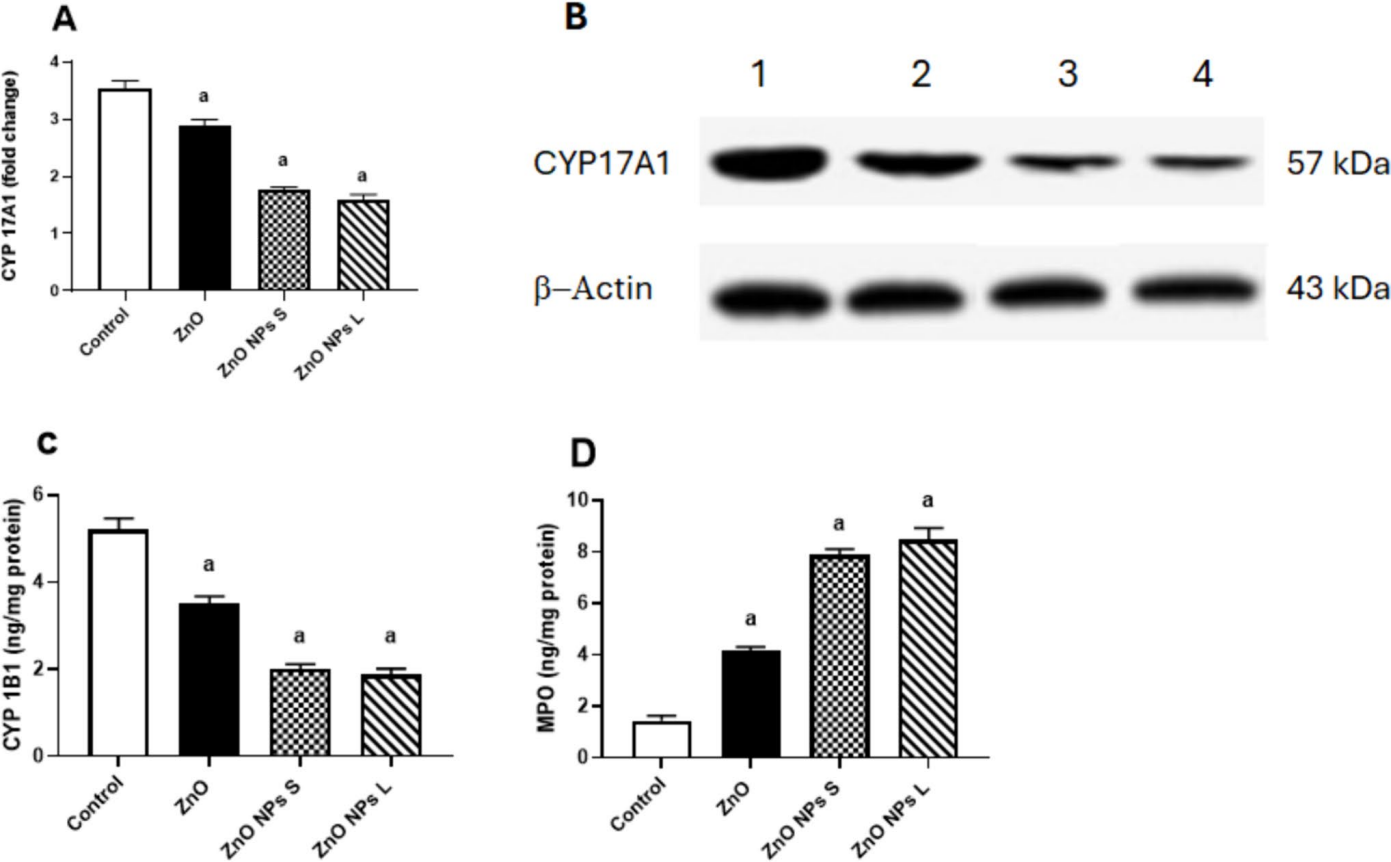
Effect of zinc oxide on the (**A**) cytochrome P450 17A1 (CYP17A1), (**B**) a representative photo of Western blot analysis of CYP17A1 (lanes 1–4 respresnt control, Zno, ZnO NPs small, and ZnO NPs large), (**C**) cytochrome 1B1 (CYP1B1), and (**D**) myeloperoxidase (MPO) in the testis. Data are expressed as Mean ± SEM, *n* = 8. ^a^ Significant difference versus control, ^b^ significant difference between different diameters. S: small size, L: large size. The bands were cropped for clarity and the whole uncropped gels can be reviewed in the supplementary file, Figures S1 and S2

All treatments with zinc oxide, whether at the macro or nano size, resulted in a significant reduction in the level of CYP17A1 in the testis homogenate compared to the control group. Treating animals with ZnO resulted in a significant reduction (*p* < 0.01, 18.7%) in the level of CYP17A1 compared to the control. Treating animals with ZnO NPs of small size caused a significant (*p* < 0.001) decrease (49.8%) in enzyme level compared to the control. The larger diameter of ZnO NPs significantly (*p* < 0.001) reduced (54.7%) the level of the enzyme compared to the control (Fig. 8A and B). Treating rats with ZnO resulted in a significant (*p* < 0.001) reduction (32.7%) in the level of CYP1B1 compared to the control. The groups treated with ZnO NPs of small size and large size showed significant (*p* < 0.001) reductions (61.5% and 64.2%, respectively) in the enzyme activity compared to the control (Fig. 8C).

Treating rats with ZnO resulted in a significant (*p* < 0.001) elevation (188.8%) in the level of MPO compared to the control. Treating animals with ZnO NPs of small size or large size caused significant (*p* < 0.001) elevations (450% and 492%, respectively) in the enzyme level compared to the control (Fig. 8D).

### Caspase 3

**Fig. 9:**
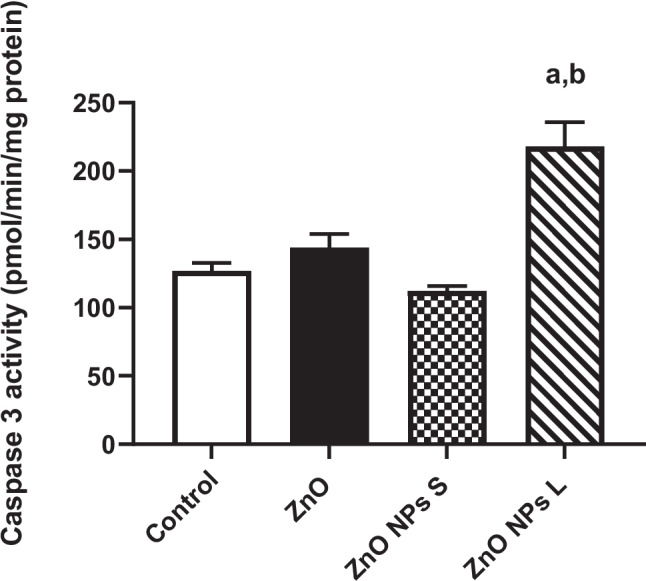
Effect of zinc oxide on the activity of caspase 3 in the testis. Data are expressed as Mean ± SEM, *n* = 8. ^a^:Significant difference versus control, ^b^:significant difference between different diameters. S: small size and L: large size

Treating animals with ZnO NPs of large size resulted in a significant (*p* < 0.001) increase (71.9%) in the activity of caspase 3 in the testis homogenate compared to the control, and a significant (*p* < 0.001) elevation (94.2%) compared to ZnO NPs of small size (Fig. 9).

### Gene Expression of *p53* and *cdk1*

**Fig. 10:**
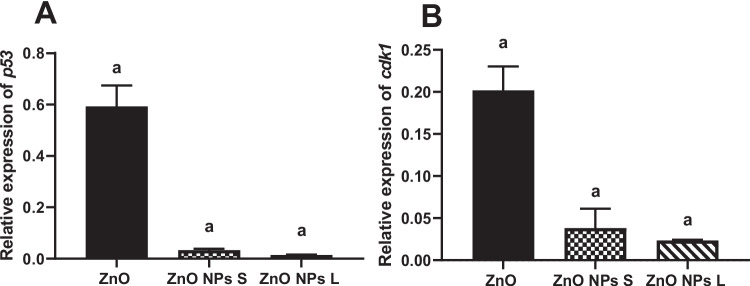
Effect of zinc oxide on gene expression of (**A**) *p53*, (**B**) *cdk1*. Data are expressed as Mean ± SEM, *n* = 5. ^a^:Significant difference versus control. S: small size and L: large size

Treating animals with ZnO caused a significant (*p* < 0.001) downregulation by 1.7-fold in the expression of *p53* in the testis tissue compared to the control group. Treating rats with ZnO NPs of small size or large size caused significant (*p* < 0.001) downregulations in the expression of *p53* (33.3-fold and 100-fold, respectively) compared to the control (Fig. 10A). Treating animals with ZnO caused a significant (*p* < 0.001) downregulation by 5.0-fold in the expression of *cdk1* in the testis tissue compared to the control group. Treating animals with ZnO NPs of small size or large size caused significant (*p* < 0.001) downregulations in the expression of *cdk1* (33.3-fold and 50-fold, respectively) compared to the control (Fig. 10B).

## Discussion

In this study, the impact of zinc oxide nanoparticles (ZnO NPs) of different diameters (40 nm and 70 nm) and ZnO macromolecules on the male reproductive system of adult rats was evaluated. Rats treated with large-sized ZnO NPs showed reductions in body weight in the 6th and 7th weeks of the experiment, with the reduction in the 7th week being statistically significant compared to the control. This weight loss exceeded 10%, indicating potential toxicity. Additionally, the same treatment significantly reduced body weight gain by approximately 37% compared to controls by the end of the experiment. Previous research has suggested that exposure to metallic nanoparticles can lead to disturbances in the gastrointestinal tract, reduced appetite, loss of fatty tissue in organs, and overall decreased body weight [22, 23].

Only the group treated with ZnO macromolecules showed a significant decrease in the relative weight of the testes compared to the control group. Other treatment groups did not exhibit significant changes in relative testes weight. Previous studies have indicated that ZnO NPs, particularly at lower doses (5 mg/kg compared to the 50 mg/kg used in this study), can significantly decrease testicular weight in rats due to processes such as apoptosis or necrosis [24, 25].

In our study, all treatments, including zinc oxide nanoparticles (ZnO NPs) of different sizes and ZnO macromolecules, did not result in any significant change in the level of zinc in the testes compared to the control group. This finding aligns with previous reports indicating that ZnO or ZnO NPs, at various doses, do not affect the testicular zinc content in rodents [26–28]. ZnO NPs are known to enter the human body and accumulate primarily in organs such as the kidneys, liver, and lungs [29]. A study using 20 nm ZnO NPs at a high dose of 350 mg/kg reported an increase in hepatic and testicular zinc content in rats, although this dose was significantly higher (7 times) than what was used in our investigation, and the nanoparticle size was smaller than those studied here [28]. The accumulation of zinc in the liver following oral administration of ZnO NPs suggests that the liver plays a crucial role in their metabolism.

In our experiment, serum alanine aminotransferase (ALT) activity did not show any significant changes, consistent with a previous study [30], indicating that liver function was not notably affected by the treatments. It has been previously reported that only levels above 50 mg/kg of ZnO NPs can induce hepatotoxicity and increase the plasma activity of ALT [31] Acid phosphatase (ACP), a lysosomal enzyme, serves as a reliable biomarker for assessing prostate and semen quality [32]. In our study, serum ACP activity significantly increased in groups treated with both ZnO and ZnO NPs of small and large diameters compared to the control group, consistent with findings from previous studies [33, 34]. ZnO NPs may penetrate the cell membrane of the prostate cells and disrupt lysosomes resulting of the release of acid phosphatase into plasma. The exact cause of this elevation is not conclusively explained, but it suggests a potential link to prostatic inflammation, although further investigation is needed to confirm this hypothesis.

In our study, sperm analysis served as a crucial tool for evaluating testicular toxicity induced by zinc oxide nanoparticles (ZnO NPs). Interestingly, the group treated with ZnO and ZnO NPs small size caused significant reductions in sperm count compared to the control. However, across both nano and macro forms of ZnO, regardless of particle diameter, there was a significant reduction in sperm motility. Notably, the smallest diameter nanoparticles caused the most pronounced decrease in motility. Additionally, all treatments led to a significant increase in the percentage of sperm abnormalities, a finding consistent with previous studies involving mice treated with ZnO NPs [27, 35]. These abnormalities in sperm morphology and motility—such as flagella abnormalities, increased residual cytoplasm, motility loss, and decreased acrosome reaction—can be attributed to several factors. These include hormonal disturbances, heightened oxidative stress, apoptosis, or compromised cell viability. Such conditions can result in significant disruptions to spermatogenesis, damage to seminiferous tubules, degeneration of Leydig cells, necrosis of spermatogenic cells, and ultimately lead to low sperm count and reduced motility [36, 37]. Given these outcomes, our study delved into these molecular pathways to better understand how ZnO NPs may impact male reproductive health at a cellular level.

Investigating the causes behind the global decline in semen quality is crucial given its significant implications [38]. One of the primary factors influencing semen quality is the disruption in the endocrine regulation of male reproductive functions. The hypothalamus-pituitary-testes axis plays a pivotal role in maintaining the normal physiology of the male reproductive system. In response to gonadotropin-releasing hormones from the hypothalamus, the anterior pituitary gland produces and releases gonadotropins, namely follicle-stimulating hormone (FSH) and luteinizing hormone (LH). These hormones act on Sertoli and peritubular cells within the seminiferous tubules, stimulating spermatogenesis. FSH binds to receptors on Sertoli cells, while LH enhances testosterone synthesis by Leydig cells. Testosterone is crucial for maintaining secondary sexual characteristics, promoting the healthy growth and development of male sex organs, and enhancing sperm morphology, epididymal functions, and motility [39].

Our study identified a significant decrease in serum free testosterone levels in groups treated with both small and large sizes of ZnO nanoparticles (NPs) compared to the control group. Previous research on the effects of ZnO NPs on testosterone levels has reported conflicting results, with some studies indicating no impact [25], while other studies reported elevation [40] or reduction [41, 42]. Additionally, both ZnO and ZnO NPs in our study resulted in significant reductions in serum LH levels compared to the control group, consistent with earlier findings [27]. This reduction in LH levels was attributed to the ability of ZnO NPs to cross the blood–brain barrier and influence the hypothalamus and pituitary gland, thereby decreasing LH secretion [43]. However, our study did not observe any significant changes in FSH levels despite its secretion by gonadotrophs. Further investigations are warranted to elucidate the mechanisms underlying the observed reduction in LH levels. These findings underscore the complex interactions between ZnO NPs and the endocrine system regulating male reproductive functions, highlighting potential mechanisms by which these nanoparticles may disrupt hormonal balance and impact reproductive health.

The decrease in LH levels observed in our study could potentially explain the reduction in testosterone production, as LH is primarily responsible for stimulating the key enzyme involved in androgen synthesis, CYP17A1. Cytochrome P450 (CYP) enzymes are essential microsomal hemoproteins that play critical roles in various biological processes such as detoxification of xenobiotics, cellular metabolism, and maintaining homeostasis. Within the testes, CYP17A1 catalyzes the conversion of pregnenolone to dehydroepiandrosterone (DHEA), which is a crucial precursor in the pathway leading to testosterone production. Specifically, CYP17A1 mediates the 17α-hydroxylation of progesterone and pregnenolone, followed by 17α,20-lyase activity leading to the synthesis of androstenedione and DHEA, respectively [44].

While CYP17A1 is primarily involved in androgen biosynthesis, another important enzyme in steroid metabolism is CYP1B1, which is expressed in tissues like the ovary, testis, and adrenal gland. CYP1B1 facilitates the 4-hydroxylation of 17beta-estradiol, playing a role in maintaining the balance between estrogen and testosterone in the testes and Leydig cells [45]. This balance is crucial for Leydig cell function and overall testicular health [46, 47]. The disruption of these enzymatic pathways, potentially induced by factors like ZnO nanoparticles (NPs), could lead to dysregulation in testosterone production and imbalance between estrogen and testosterone levels. This dysregulation may contribute to the observed impacts on male reproductive health, including alterations in sperm parameters and hormonal profiles observed in our study and others investigating the effects of ZnO NPs on male reproductive function. Further research is necessary to fully elucidate the mechanisms by which ZnO NPs affect these crucial enzymatic pathways and their implications for male reproductive health.

Our study revealed significant reductions in the levels of CYP17A1 and CYP1B1 in the testes following exposure to zinc oxide (ZnO) at both macro and nano sizes, compared to the control group. This finding aligns with previous research [48], highlighting that ZnO nanoparticles (NPs), particularly at the nano size, induced the most pronounced decrease in these key enzymes involved in steroid metabolism. The current understanding of how Zn nanoparticles influence CYP450 enzymes is limited. Based on our findings, we propose a mechanism where ZnO NPs reduce LH levels, which subsequently diminishes the expression and activity of CYP17A1 and CYP1B1. These enzymes are critical for the biosynthesis of testosterone and the maintenance of the estrogen-androgen balance within the testes and Leydig cells [44, 45, 47]. Disruption of these pathways likely contributes to the observed reduction in testosterone synthesis, as well as disturbances in the estrogen-androgen ratio.

The second potential explanation for the deteriorations observed in sperm analysis is oxidative stress induced by nanotoxicity. Certain nano-metal oxides, including zinc oxide nanoparticles (ZnO NPs), can stimulate the production of reactive oxygen species (ROS). These ROS can lead to DNA damage, oxidative stress, and subsequent alterations in cellular functions such as motility, inflammation, viability, and apoptosis. To evaluate oxidative stress in our study, we measured several markers: glutathione (GSH) as an indicator of intracellular antioxidant capacity, superoxide dismutase (SOD) which breaks down harmful superoxide radicals, and malondialdehyde (MDA) as a marker of lipid peroxidation induced by oxidative stress [49].

Interestingly, our findings showed divergent results regarding oxidative stress markers in testis homogenates treated with ZnO NPs of different sizes. Treatment with small-sized ZnO NPs significantly elevated SOD activity compared to the control group, indicating a response to increased oxidative stress. Conversely, treatment with large-sized ZnO NPs led to a significant decrease in MDA levels compared to the control, suggesting a protective effect against lipid peroxidation and potential preservation of cell membrane integrity. Previous research has reported conflicting results regarding the impact of ZnO NPs on oxidative stress in the testes. Some studies have shown that ZnO NPs elevate antioxidant levels and reduce oxidative stress [50, 51], while others have demonstrated impairment of the blood-testes barrier and increased oxidative stress [49, 52]. Additionally, some studies have reported no significant changes in SOD, GSH, and MDA levels following ZnO NP exposure [53]. Based on our observations, we hypothesized that inflammation may play a crucial role in the deteriorations observed in semen analysis. Inflammation, as assessed by markers such as myeloperoxidase (MPO) activity, can be a critical factor contributing to oxidative stress and subsequent damage to sperm quality and reproductive function.

Myeloperoxidase (MPO) is an enzyme involved in oxidative stress and inflammation processes. It catalyzes the conversion of hydrogen peroxide to hypochlorous acid, which can activate pathways leading to cellular senescence or apoptosis [54, 55]. Additionally, MPO converts tyrosine to tyrosyl radical using hydrogen peroxide as an oxidizing agent, generating cytotoxic compounds that can induce oxidative damage [56]. Elevated MPO levels have been associated with increased risks of cardiovascular mortality and severity of coronary artery disease [57].

In our study, we found that treatment with both zinc oxide (ZnO) and zinc oxide nanoparticles (ZnO NPs) of small and large diameters significantly increased MPO activity in the testis compared to the control group. This elevation indicates severe inflammation and suggests potential pathways towards apoptosis in the testicular tissue. Contrary to the increase in MPO activity, we observed a significant rise in superoxide dismutase (SOD) activity only in the group treated with small-sized ZnO NPs, with no change in glutathione (GSH) levels. This discrepancy suggests that while oxidative stress pathways involving superoxide anions or ROS may not be directly involved in the inflammatory response indicated by MPO elevation, other inflammatory pathways cannot be ruled out. Therefore, to further understand the mechanisms involved, we investigated markers of apoptosis and cell viability in the testis.

Caspase-3 is a critical protease involved in apoptosis, activated through various pathways including DNA damage [58]. Our results revealed that while all treatments increased myeloperoxidase (MPO) activity, indicating inflammation, only ZnO NPs of large size significantly elevated caspase-3 activity in the testis. This finding suggests induction of apoptosis, consistent with previous research [22, 59, 60].

The tumor suppressor protein p53 plays a pivotal role in maintaining genomic stability by regulating cell cycle arrest, senescence, and apoptosis. Mutations in p53 are prevalent in many cancers, underscoring its importance in cellular health and disease prevention [61]. In our study, both ZnO and ZnO NPs, irrespective of their size, significantly downregulated p53 gene expression in the testis compared to the control group. Notably, ZnO NPs induced more pronounced reductions in *p53* expression than macromolecules, indicating potential disruption in cellular responses to DNA damage and stress. Cdk1, a member of the cyclin-dependent kinase family, is crucial for driving cells through the cell cycle phases, particularly from the S phase to the G2/M phase transition [62]. Our findings demonstrated significant down-regulation of *cdk1* expression in the testis following exposure to ZnO or ZnO NPs, aligning with studies where ZnO NPs were shown to arrest cells in the S phase due to *cdk1* down-regulation [63, 64]. Contrary to our findings, some studies have reported elevated *p53* expression in response to ZnO NPs [52, 65], indicating variability in cellular responses possibly influenced by dosage, exposure duration, or experimental conditions.

## Conclusion

Our study demonstrated that zinc oxide nanoparticles (ZnO NPs) exerted significant adverse effects on the male reproductive system in rats. Specifically, ZnO NPs reduced luteinizing hormone (LH) levels, which subsequently led to decreases in the levels of CYP17A1 and CYP1B1 enzymes involved in testosterone synthesis. This hormonal disruption resulted in reduced testosterone production. Additionally, ZnO NPs induced testicular inflammation and decreased cell viability. These impacts were reflected in increased sperm abnormalities and decreased sperm motility, indicating compromised semen quality. Importantly, the toxicity induced by ZnO NPs was more pronounced compared to ZnO macromolecules. Furthermore, the large-diameter ZnO NPs demonstrated greater toxicity than the small-sized particles. Overall, our findings underscore the potential reproductive toxicity of ZnO nanoparticles, highlighting the need for further research to better understand the mechanisms underlying these effects and to develop strategies for minimizing nanoparticle-related health risks in both occupational and consumer settings.

## Supplementary Information

Below is the link to the electronic supplementary material.

Supplementary file1 (DOCX 195 KB)

## Data Availability

All data are presented in the manuscript.
