# Supplementary material for: Zinc Oxide Nanoparticles Induced Testicular Toxicity Through Inflammation and Reducing Testosterone and Cell Viability in Adult Male Rats
Source: Biol Trace Elem Res. 2024 Aug 12;203(4):1934–48. doi: 10.1007/s12011-024-04330-1 (PMC11919946; doi:10.1007/s12011-024-04330-1)
Supplement: Supplementary file 1 — Supplementary file1 (DOCX 195 KB) [file 12011_2024_4330_MOESM1_ESM.docx]

**Zinc oxide nanoparticles induced testicular toxicity through inflammation and reducing testosterone and cell viability in adult male rats**

**Dina H. Ahmed, Nadia M. El-Beih, Enas A. El-Hussieny, Wael M. El-Sayed^*^**

Department of Zoology, Faculty of Science, Ain Shams University, Abbassia 11566, Cairo, Egypt

**^*^Corresponding author:**

Wael M. El-Sayed,

Department of Zoology, Faculty of Science, Ain Shams University, Abbassia 11566, Cairo, Egypt

Email; [wael_farag@sci.asu.edu.eg](mailto:wael_farag@sci.asu.edu.eg), [waelelhalawany@hotmail.com](mailto:waelelhalawany@hotmail.com)

ORCID: 0000-0002-3622-1417

Running title: Zinc oxide nanoparticles caused testicular toxicity.

**Western blot procedure**

**Protein extraction procedure**

The ReadyPrepTM protein extraction kit (total protein) provided by Bio-Rad Inc (Catalog #163-2086) was employed according to manufacturer’s instructions. Bradford Protein Assay Kit (SK3041) for quantitative protein analysis was provided by Bio basic Inc (Markham Ontario L3R 8T4 Canada). A Bradford assay was performed according to manufacture instructions to determine protein concentration in each sample. A 20 μg protein concentration of each sample was then loaded with an equal volume of 2x Laemmli sample buffer containing 4% SDS, 10% 2-mercaptoehtanol, 20% glycerol, 0.004% bromophenol blue and 0.125 M Tris HCl. The pH was adjusted to 6.8. Each previous mixture was boiled at 95°C for five min to ensure denaturation of protein before loading on polyacrylamide gel electrophoresis.

**Protein separation by electrophoresis**

Samples were separated on sodium dodecyl sulfate-polyacrylamide gel electrophoresis (SDS-PAGE) using TGX Stain-Free™ FastCast™ Acrylamide Kit, which was provided by Bio-Rad Laboratories inc Cat # 161-0181. The SDS-PAGE TGX Stain-Free FastCast was prepared according to manufacturer’s instructions.

**Protein blotting (transfer of proteins from the gel to the membrane)**

The gel was assembled in transfer sandwich as following from below to above (filter paper, PVDF membrane, gel, and filter paper). The sandwich was placed in the transfer tank with 1x transfer buffer, which is composed of 25 mM Tris and 190 mM glycine and 20% methanol. Then, the blot was run for 7 min at 25 V to allow protein bands transfer from gel to membrane using BioRad Trans-Blot Turbo.

**Blocking the membrane**

The membrane was blocked in tris-buffered saline with Tween 20 (TBST) buffer and 3% bovine serum albumin (BSA) at room temperature for an hour. The components of blocking buffer were 20 mM Tris pH 7.5, 150 mM NaCl, 0.1% Tween 20 and 3% BSA.

**Incubation with the primary antibody**

Primary antibodies of CYP17A1 were diluted in TBST. Incubation was performed overnight in primary antibody solution against the blotted target protein at 4°C. The blot was rinsed 3–5 times for five minutes with TBST. Incubation was done in the horse radish peroxidase-conjugated secondary antibody (Goat anti-rabbit IgG- HRP-1mg Goat mab -Novus Biologicals) solution against the blotted target protein for an hour at room temperature. The blot was rinsed 3–5 times for five minutes with TBST.

**Imaging and data analysis quantitation**

The chemiluminescent substrate (ClarityTM Western ECL substrate Bio-Rad cat#170-5060) was applied to the blot according to the manufacturer’s recommendation. Briefly, equal volumes were added from solution A (Clarity western luminal/enhancer solution) and solution B (peroxidase solution). The chemiluminescent signals were captured using a CCD camera-based imager. Image analysis software was used to read the band intensity of the target proteins against control sample beta actin (housekeeping protein) by protein normalization on the ChemiDoc MP imager.


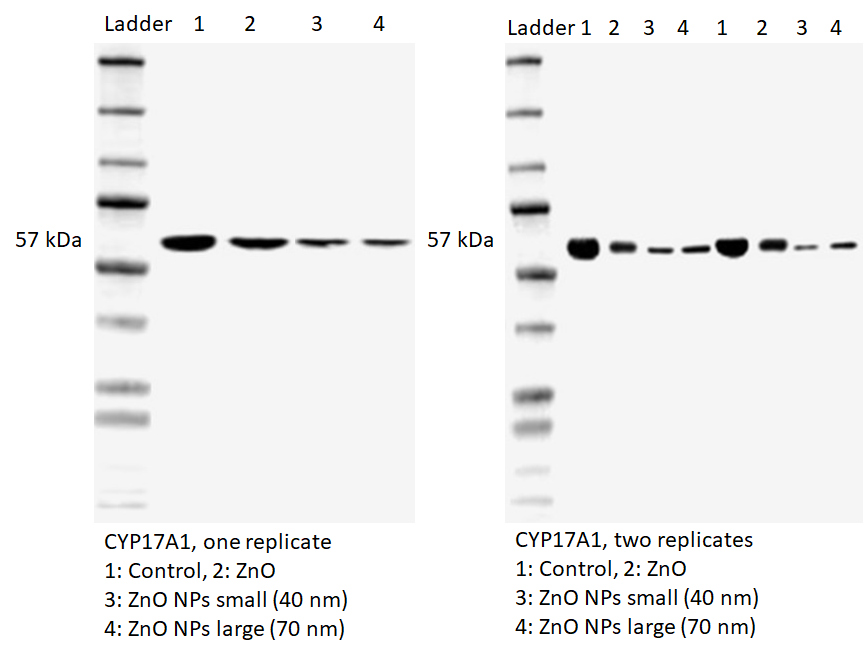


**Figure S1: The whole uncropped gels for** Western blot analysis of CYP17A1 (n = 3)


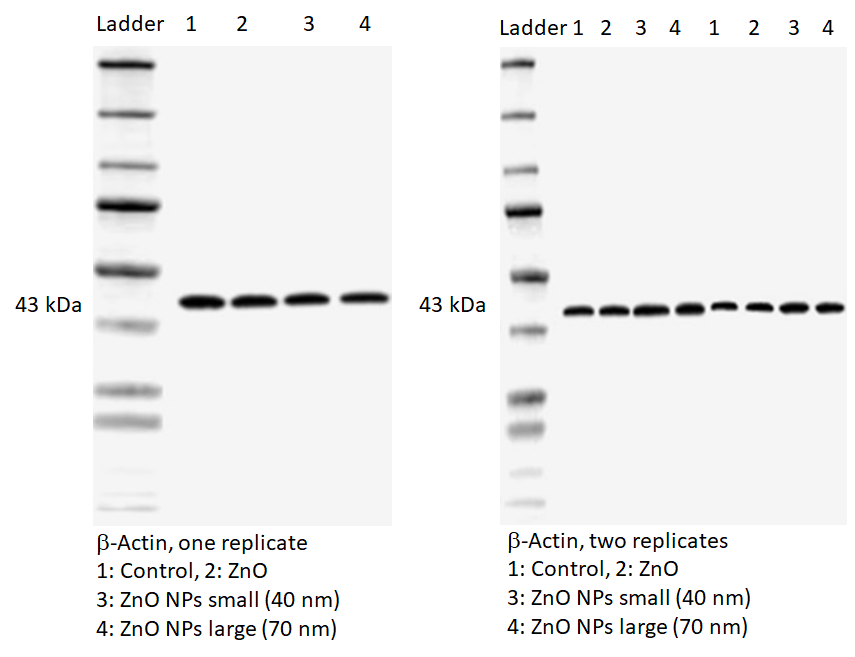


**Figure S2: The whole uncropped gels for** Western blot analysis of β-actin (n = 3)

**Standard curves of biochemical parameters measured**

Figure S3: Standard curve of free testosterone

Figure S4: Standard curve of luteinizing hormone

Figure S5: Standard curve of FSH

Figure S6: Standard curve of ALT

Figure S7: Standard curve of total protein

Figure S8: Standard curve of CYP1B1

Figure S9: Standard curve of Myeloperoxidase

**Table S1: Animal diet ingredients (g/kg)**

| **Ingredient** | **Content (g/kg)** |
| --- | --- |
| **Corn starch** | 480.1 |
| **Casein** | 189.5 |
| **Maltodextrin** | 118.4 |
| **Sucrose** | 65.2 |
| **Cellulose** | 47.4 |
| **Soybean oil** | 23.7 |
| **Lard** | 18.9 |
| **Potassium citrate** | 15.6 |
| **Dicalcium phosphate** | 12.3 |
| **Mineral mix** | 9.5 |
| **Vitamin mix** | 9.5 |
| **Calcium carbonate** | 5.2 |
| **L-Cystine** | 2.8 |
| **Choline bitartrate** | 1.9 |
| **Total** | **1000** |

The diet provides 3738 kcal/kg
